# Within- and between-population comparisons suggest independently acting selection maintaining parallel clines in Scots pine (*Pinus sylvestris*)

**DOI:** 10.1093/evlett/qrad054

**Published:** 2023-11-27

**Authors:** Sonja T Kujala, Komlan Avia, Timo A Kumpula, Hanni Kärkkäinen, Juha Heikkinen, Katri Kärkkäinen, Outi Savolainen

**Affiliations:** Natural Resources Institute Finland, Oulu, Finland; University of Oulu, Ecology and Genetics, Oulu, Finland; Université de Strasbourg, INRAE, SVQV UMR-A 1131, Colmar, France; University of Oulu, Ecology and Genetics, Oulu, Finland; Natural Resources Institute Finland, Jokioinen, Finland; Natural Resources Institute Finland, Helsinki, Finland; Natural Resources Institute Finland, Oulu, Finland; University of Oulu, Ecology and Genetics, Oulu, Finland

**Keywords:** budset timing, fall frost injury, first-year height, genetic correlation, local adaptation, selection gradient

## Abstract

Parallel clines in traits related to adaptation in a species can be due to independent selection on a pair of traits, or due to selection in one trait resulting in a parallel cline in a correlated trait. To distinguish between the mechanisms giving rise to parallel adaptive population divergence of multiple traits along an environmental gradient we need to study variation, correlations, and selective forces within individual populations along the gradient. In many tree species, budset timing (BST) forms a latitudinal cline, and parallel clinal variation is also found in other seedling traits, such as first-year height (FYH) and fall frost injury (FFI). In this study, we set up a common garden experiment with open pollinated progeny from natural populations of Scots pine (*Pinus sylvestris*), with one large sample from single population (500 families) and smaller samples from across a latitudinal gradient. BST, FYH and induced FFI were first measured in a greenhouse. The seedlings were then planted in the field, where survival and height were measured at the age of 9 years as fitness proxies. We compared between- and within-population variation and genetic correlations of these three seedling traits, and estimated selection gradients at the family level in our main population, taking into account the potential effects of seed weight. Between-population genetic correlations between seedling traits were high (0.76–0.95). Within-population genetic correlations in the main population were lower (0.14–0.35), as in other populations (0.10–0.39). Within population, extensive adaptive variation persists in the seedling traits, in line with rather weak selection gradients, yet maintaining the clines. Although our sampling does not cover the whole cline equally, the results suggest that the individual clines in these traits are maintained by largely independently acting selection, which results in fewer constraints in adaptation under changing climate.

## Introduction

When environmental conditions change gradually over an area, for example, latitudinally, adaptation can be reflected as clinal patterns where population means of adaptation-related traits track local optima (Felsenstein, 1977). Clinal variation in many traits of plants and animals has been observed throughout centuries of research (Endler, 1977). Where overall genomic variation suggests little or no clinal differentiation, the clines likely have been formed by the action of natural selection (Chen et al., 2014; Kujala & Knürr et al., 2017; Mimura & Aitken, 2007; Savolainen et al., 2007; Viherä-Aarnio et al., 2005). When populations along the cline are connected through current gene flow, clines reflect a balance between selection and gene flow (Slatkin, 1973, 1978).

Many different traits within a species may display similar clines, raising the question whether selection has acted independently on the traits, or selection on one trait has resulted in correlated clines in other traits through physiological constraints and/or pleiotropy and linkage disequilibrium (indirect selection; Guillaume, 2011; Lande, 1979; Merilä & Björklund, 1999). Kingsolver et al. (2001) did not find much evidence of indirect selection. Geber and Griffen (2003), in contrast, concluded that indirect selection effects are common among plant studies. To resolve these issues, it is important that the covariation and the effects of selection are also studied within population. Between- and within-population correlations in many adaptive traits have been reported (see Howe et al., 2003 for review on conifers), but comparisons of these two levels of correlations are needed in natural populations sampled along clines.

Genetic models of clinal variation assume that each population evolves toward a different optimum value of the trait through diversifying selection, while stabilizing selection keeps each local population concentrated around its local optimum (Barton, 1999; Slatkin, 1978). Within population, recombination, gene flow, and mutation bring in new variation that selection acts on (Walsh & Lynch, 2018). Selection gradients for the individual traits—and selection on the correlation itself—can be measured via a multivariate analysis (Arnold et al., 2008; Geyer et al., 2007; Kingsolver & Pfennig, 2007; Lande & Arnold, 1983; Phillips & Arnold, 1989). A prospective study, where individuals of a cohort are characterized for the traits of interest early on, and then monitored for fitness, allows catching part of the lifetime selection.

Scots pine (*Pinus sylvestris*) is a widespread wind pollinated boreal conifer with a large effective population size (Mirov, 1967), that shows very little differentiation of genomic markers between populations (Pyhäjärvi et al., 2019; Savolainen et al., 2007; Tyrmi et al., 2020). Seedling traits, however, show clinal variation in common garden conditions (Aho, 1994; Kujala & Knürr et al., 2017; Mikola, 1982; Notivol et al., 2007). In many other tree species as well, budset timing (BST) at the end of the first growing season shows a clear latitudinal cline in population means (Alberto et al., 2013; Howe et al., 2003). As fall approaches, northern populations in common gardens set their buds on average earlier (in longer days) than southern populations, an apparent adaptation to avoid cold damage in the fall. A cline is also observed in first-year height (FYH), as southern seedlings grow taller than the northern ones in common gardens (Alberto et al., 2013; Notivol et al., 2007). In natural conditions, the size of the seedling can be an indicator of general vigor and competitive ability in dense populations and among other vegetation (for Scots pine, see Jäghagen & Albrektson, 1996; Johansson et al., 2015). Fall frost injury (FFI) also shows a clinal pattern (Aho, 1994; Howe et al., 2003; Hurme et al., 1997), and these three traits are highly correlated across divergent populations (Hurme et al., 1997; Mikola, 1980).

The nature of genetic correlations and selection pressures in these traits is highly relevant in a warming climate; constraints due to correlations can influence the ability of trees to adapt via changes in the timing of annual growth (Aitken et al., 2008; Benito Garzón et al., 2011; Bradshaw & Holzapfel, 2001; Etterson & Shaw, 2001; Oleksyn et al., 1998; Saikkonen et al., 2012; Savolainen et al., 2004). Here we examine the interdependence of clines. We make two distinct predictions: (a) Under the scenario of independently formed clines, we expect to see high between population, but low within-population correlations between traits. Furthermore, selection gradients within population are expected to be significant for each independent trait, and selection for between trait correlation not significant. (b) If parallel clines form as a result of indirect selection, we expect high correlations both between and within populations. For the trait under predominantly indirect selection, the selection gradient is expected to be low. We compared the correlation patterns between and within populations and characterized intrapopulation selection gradients. Although our analysis is mainly from a single large population and further work in other parts of the clines is needed, our results offer support to the hypothesis that the different seedling clines have not formed solely because of correlated, indirect selection responses.

## Methods

### Material

**Figure 1.**
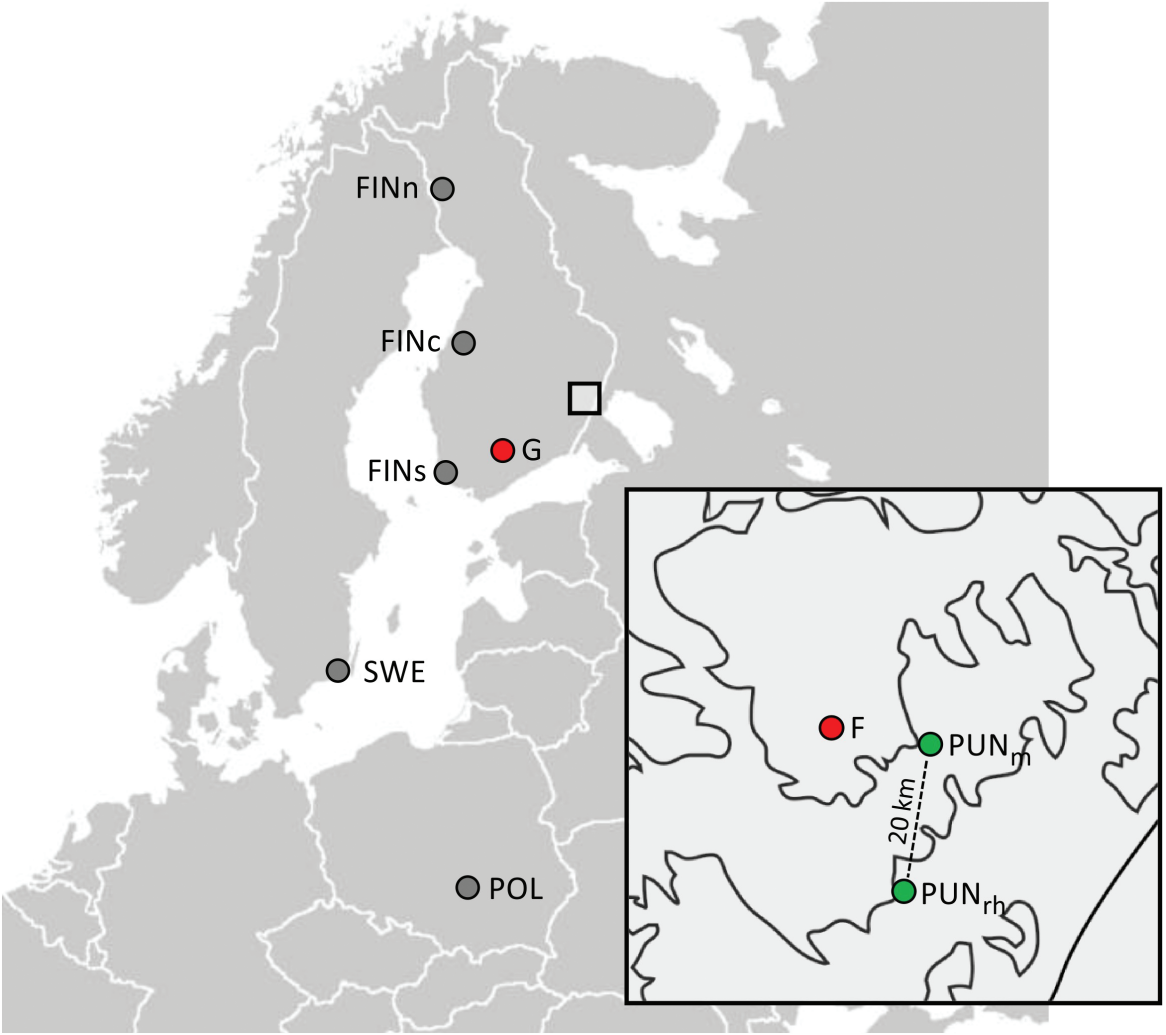
Origins of genetic material used in this study, and locations of study sites (greenhouse, G, and field site, F, in inset). Punkaharju population ( in inset) consists of two close-by subpopulations in Eastern Finland, Ranta-Halola (PUN_rh_) and Mäkrä (PUN_m_). Latitudinal populations: POL = Poland, SWE = Sweden, FINs = Southern Finland, FINc = Central Finland, FINn = Northern Finland.

Our focal population consists of naturally regenerated stands in the Evoltree Intensive Study Site in Punkaharju (EVOLTREE, n.d.), southeastern Finland. Open pollinated seed from 500 trees were sampled in two areas roughly 20 km apart with a minimum distance of 20 m between most pairs of trees to avoid close relatedness. About 380 trees were selected in Ranta-Halola (61°39ʹN, 29°17ʹE), and 120 trees in the slightly more northern Mäkrä (61°50ʹN, 29°23ʹE) site (Table 1; Figure 1), assuming that two naturally regenerated Scots pine stands 20 km apart represent the same gene pool, and that the trees in both locations have adapted to similar environmental conditions. The age of the mother trees (estimated from the number of tree rings at breast height) varied between 33 and 145 years. For each mother tree, 24 open pollinated seedlings were sown in a greenhouse experiment. We expect open pollinated offspring to be mostly half-sibs (Kärkkäinen & Savolainen, 1993; Muona & Harju, 1989; Squillace, 1974).

**Table 1.** Origins and sample sizes of the plant material used in this study.

Population	Country	Latitude	Longitude	No. of families
PUN	Finland	61°39ʹN–61°50ʹN	29°17ʹE–29°23ʹE	500
FINn	Finland	67°11ʹN	24°03ʹE	10
FINc	Finland	63°45ʹN	24°05ʹE	10
FINs	Finland	60°52ʹN	21°20ʹE	10
SWE	Sweden	56°28ʹN	15°55ʹE	10
POL	Poland	50°41ʹN	20°05ʹE	10

*Note.* FINc = Central Finland; FINn = Northern Finland; FINs = Southern Finland; POL = Poland; PUN = Punkaharju; SWE = Sweden.

In addition, 10 open pollinated families from five natural populations covering latitudes ~50°N–67°N were included, and hereafter called latitudinal populations (Table 1; Figure 1). The number of half-sib seedlings per family in these populations was 12. We settled for a smaller sample size in these latitudinal populations, as the clinal variation has been characterized in many previous studies with larger data.

### Greenhouse experiment and measurements

During June 9th–13th, 2008, a greenhouse experiment was established in Loppi, Finland (60°37ʹN, 24°25ʹE; Figure 1). The open pollinated seed of 550 mother trees were sown in disinfected Plantek 49F seedling trays filled with Kekkilä M6 W peat. The greenhouse had ambient temperature and light conditions. Seedlings were watered regularly. The material was fully randomized within six blocks; four seedlings per family for Punkaharju; and two for latitudinal populations were grown within each block. The FYH (in mm) of each seedling was measured with a caliper from the lowest needle to the top of the seedling from August 4th to October 24th until there was no change for three consecutive weeks. BST was recorded as days from sowing to the formation of the terminal bud (scored visually as when the bud was completely visible). Each block was scored for budset and height once a week (blocks 1–5 on consecutive days, block 6 in five parts on 5 consecutive days). Data on seedlings that died already during the greenhouse experiment or showed signs of not being healthy were excluded.

To obtain estimates for FFI, a similar greenhouse experiment with the same families and seedlots was repeated in 2010. About 12 seedlings per family from Punkaharju, and six seedlings per family for the latitudinal populations were sown between June 8th and 10th, and grown in a similar manner as in 2008, in three blocks. As the daylength and temperature in the greenhouse decreased (following outdoor conditions), growth cessation and cold acclimation were triggered. In the week of October 4th–8th, the seedlings were taken to a cold chamber. The cold treatment followed the protocol in, for example, Aho (1994). Preliminary testing (data not shown) for finding the exact treatment conditions was first done to ensure that there was variation in freezing damage. Seedlings were first sprayed with distilled water to prevent excessive super-cooling before initiation of cold exposure. The freezing test conditions were: Incubation in +5 °C for 1 hr; cooling at the rate of 3 °C hr^−^1; freezing at the target temperature of −20° C for 4 hr; warming at the rate of 3 °C hr^−^1; incubation at +5 °C for 6 hr. After treatment the seedlings were kept in a greenhouse under natural light conditions. Two weeks after the exposure to frost, each seedling was visually scored for damage indicated by chlorophyl breakdown that is, the percentage of browned needles area. Five ordinal classes for FFI assessments were used as follows: 0 = no damage, 1 = 25% damage, 2 = 50% damage, 3 = 75% damage, and 4 = dead. FYH measurements and BST are not reported here for the 2010 experiment as that data was truncated (seedlings were taken to the cold treatment before all had ceased growth and set bud).

### Field experiment and measurements

We set up a field experiment in which we measured the survival and height of the seedlings at the age of 9 years. Height at this age is not merely a measure of growth capacity, but also of the general health of the tree, perhaps even of future growth and reproductive potential and is often used as fitness proxy (Savolainen et al., 2007; Younginger et al., 2017). Half of the seedlings of the 2008 greenhouse experiment (three blocks) were used to establish this field experiment. All 500 + 50 families were represented. For the first winter, these seedlings were placed in a controlled cold room to overwinter, covered with tarpaulin. After overwintering they were transferred to a nursery garden close to the origin of the focal population, where they were kept for one full growing season. All surviving seedlings were planted in the final study field in Tihvii (61°52ʹN, 29°17ʹE) (Figure 1) in early June 2010. The study field covers 2 km^2^ of flat dry boreal forest soil. The seedlings were fully randomized within 10 field blocks and placed roughly 2 m apart. Each family was represented by one seedling within each block. These 10 blocks were surrounded by a minimum of one row of buffer seedlings. Survival in 2010 was recorded at the time of planting and subsequent survival check was performed during fall 2011.

The traits measured on the 9-year-old trees in 2017 were survival and tree height. Trees were scored as dead (0) or alive (1). Missing trees were scored as dead. Trees which could not be identified were excluded. Living trees were measured in 5 cm intervals. In branched trees only the longer branch was measured.

### Seedling trait variance, family estimates, heritabilities

Variance in seedling traits was examined both at the level of individual seedlings, and at family level. For Punkaharju, a linear mixed model


$$\begin{array}{l} Y_{ijq}=\;m\;+\;b_{i}+\;f_{j}+\;e_{ijq} \end{array}$$ (1)

where *Y*_*ijq*_ is the trait value of the *q*th seedling in *i*th block belonging to family *j*, *m* is the overall mean, *b*_*i*_ is the fixed effect of the *i*th block, *f*_*j*_ is the random effect of the *j*th family, and *e*_*ijq*_ the random error term, was fitted with REML (restricted maximum likelihood) using the function lme in R (R Core Team, 2022, version 4.2.2) in package “nlme” (Pinheiro et al., 2020, version 3.1-148). Family estimates were calculated as family BLUPs (best linear unbiased predictors) from *Equation 1*, centered around the intercept (mean). *Equation 1* was also used for FFI, as the underlying phenotype is continuous in spite of the ordinal scale scoring.

Additive genetic variances (*V*_A_) for the traits were estimated from the above model as 4 times the variance attributable to the family effect. Narrow sense heritabilities were calculated as (*V*_A_) divided by the total variance. Standard errors for heritabilities were calculated as square root of Dickerson approximation for variance of heritability (Dickerson, 1969). *V*_A_ and heritability were estimated only in the Punkaharju population as the latitudinal population samples were considerably smaller. Our estimates of *V*_A_ and heritability assume that the progenies consist of half-sibs only, that mother trees are unrelated, and that there are no maternal environmental or genetic effects. Violations of these assumptions could inflate the estimates. However, full sibs are rare in Scots pine and maternal trees of this population have very low relatedness (Harju & Muona, 1989; Niskanen et al. in review). Maternal effects through SW were examined in the Supplementary material.

Student’s two-sided *t*-test was used to test for differences in mean trait values between the subpopulations Ranta-Halola and Mäkrä at the level of family estimates. Effects of early mortality on seedling trait distributions were examined in the Supplementary material.

### Phenotypic and genetic correlations

Phenotypic correlations were calculated as Pearson correlation of the individual seedling values in Punkaharju population. Within-population genetic correlations were estimated in Punkaharju population as Pearson correlation of the family estimates from *Equation 1*. For the latitudinal populations, a model


$$\begin{array}{l} Y_{ijq}=\;m\;+\;b_{i}+\;p_{k}+\;f_{j}+\;e_{ijq} \end{array}$$ (2)

was fitted. This is similar to *Equation 1* except for having population *k* as an additional fixed factor. Between-population correlations were calculated (a) as Pearson correlations of population wise family estimates *m* + *f*_*j*_*+ p*_*k*_ (*n* = 50) and (b) with population means of family estimates (*n* = 5). We also estimated a second set of within-population correlations using the combined family estimates (*n* = 50) of the latitudinal populations, *m* + *f*_*j*_. Multicollinearity between the traits was checked using the Punkaharju data by computing variance inflation factors with R package “car” (Fox & Weisberg, 2019). Maternal environmental and genetic effects could increase the family component of variation. These genetic correlations can thus not be considered strictly additive.

### Estimation of selection gradients

Estimates of the strength of directional selection (average slope of fitness surface) and quadratic selection (curvature of relative fitness surface near population mean) can be acquired from a multiple regression of relative fitness on the traits of interest (Arnold et al., 2008; Kingsolver & Pfennig, 2007; Lande & Arnold, 1983; Phillips & Arnold, 1989). The method corrects for the possible correlations among the measured traits, and thus selection gradients describe only the direct effects of selection on a particular trait, compared to the selection differential which encompasses both the direct and indirect effects. Here, we analyzed selection gradients in the Punkaharju population at the family level (see Rausher, 1992) with a multiple regression model including all seedling traits (BST, FYH, and FFI) and seed weight (SW). Family estimates from *Equation 1* were used as an input for the seedling traits, and the weight of 200 seeds from the respective seedlots as seed weight. These four traits were first standardized by dividing the values with their standard deviations. Absolute fitness for each family was calculated in two ways: (a) as a family estimate of height in 2017 (age 9 at the field site) with *Equation 1*, also including the dead seedlings with height 0, and (b) as a proportion of surviving seedlings in each family. Relative fitness was then obtained by dividing with mean absolute fitness. Selection analysis was repeated by omitting seedlings that died before planting in the field (Supplementary material).

As our data were not strictly multivariate normal (Madria’s test in R package “MVN”; Korkmaz et al., 2014), we calculated the gradients with a two-step process (Lande & Arnold, 1983; Phillips & Arnold, 1989): Estimates for directional selection were first obtained from a model consisting only of the linear terms,


$$\begin{array}{l} w=\alpha +\underset{i=1}{\overset{4}{\sum }}\;\beta_{i}f_{i}+\epsilon , \end{array}$$ (3)

where w is relative fitness, $f_{i}$ family estimate of trait i, α an intercept, and $\beta_{i}$ the directional selection gradient of trait i. The nonlinear selection gradients were then estimated using a model containing also the second-order terms,


$$\begin{array}{l} w=\alpha +\underset{i=1}{\overset{4}{\sum }}\;\beta_{i}^{*}f_{i}+\underset{i=1}{\overset{4}{\sum }}\;\frac{1}{2}\gamma_{ii}f_{i}^{2}+\underset{i=1}{\overset{4}{\sum }}\;\underset{k>i}{\overset{4}{\sum }}\;\gamma_{ik}f_{i}f_{k}+\epsilon , \end{array}$$ (4)

where $\gamma_{ii}$ gives the stabilizing/disruptive selection gradient of trait i, and $\gamma_{ik}$ the correlational selection gradient for traits i and k. In both cases, parameters were estimated using R function lm(). (Note that the function returns the quadratic coefficients as ½ $\gamma_{ii}$, and hence must be doubled [see Stinchcombe et al., 2008]).

Model in *Equation 4* indicated statistically significant correlational selection for some pairs of traits, possibly leading to biased estimates for the stabilizing/disruptive selection gradients. Therefore, we also performed a canonical analysis using mutually independent index traits, following Blows and Brooks (2003); Brooks et al. (2005); Phillips and Arnold (1989); and Simms (1990). The canonical analysis is based on an Eigen decomposition of the symmetric matrix


$$\Gamma =\left[\begin{array}{cccc} \gamma_{11} & \frac{1}{2}\gamma_{12} & \frac{1}{2}\gamma_{13} & \frac{1}{2}\gamma_{14}\\ \frac{1}{2}\gamma_{12} & \gamma_{22} & \frac{1}{2}\gamma_{23} & \frac{1}{2}\gamma_{24}\\ \frac{1}{2}\gamma_{13} & \frac{1}{2}\gamma_{23} & \gamma_{33} & \frac{1}{2}\gamma_{34}\\ \frac{1}{2}\gamma_{14} & \frac{1}{2}\gamma_{24} & \frac{1}{2}\gamma_{34} & \gamma_{44} \end{array}\right]$$

consisting of the nonlinear gradients from *Equation 4*, $\Gamma =M\Lambda M^{T}$, where **M** is an orthonormal matrix with the eigenvectors **m**_*i*_ of Γ as its columns, and Λ a diagonal matrix of the corresponding eigenvalues $\lambda_{i}$. Now we can write *Equation 4* in matrix form, expand with $MM^{T}=I$, reorganize and rename to obtain


$$\begin{array}{l} w=\alpha +\left(f^{T}M\right)\left(M^{T}\beta^{*}\right)+\left(f^{T}M\right)M^{T}\Gamma M\left(M^{T}f\right)=\alpha +z^{T}\theta +z^{T}\Lambda z, \end{array}$$ (5)

where $z=M^{T}f$ represents values of the new index variable, $\theta =M^{T}\beta^{*}$ the vector of directional selection gradients with respect to the index variables and the eigenvalues $\lambda_{i}$ the stabilizing/disruptive selection gradients for index trait i. The relationship between original and index traits can be ascertained as trait’s loadings in the eigenvectors **m**_*i*_. Significance levels for the canonical gradients were obtained by using the index variables in a quadratic regression model similar to *Equation 4*. The stationary point of the selection surface with respect to the index variables was determined by setting the derivative of *Equation 5* to zero and solving for $z^{*}=\;-\theta /2\Lambda$.

## Results

### Seedling trait variation

**Figure 2.**
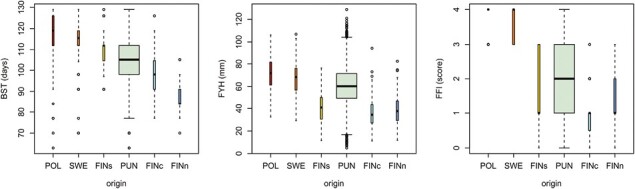
Phenotypic variation in seedling traits measured in the greenhouse during the first growing season. Plotted values are the individual seedling measurements. BST and FYH were measured in 2008 trial, FFI in 2010 trial. POL = Poland, SWE = Sweden, FINs = Southern Finland, PUN = Punkaharju, FINc = Central Finland, FINn = Northern Finland. BST = budset timing, FYH = first-year height, FFI = fall frost injury.

Within-population variation in seedling traits was extensive, especially in Punkaharju population with 500 half-sib families. Population trait means form distinct clines, while population distributions overlapped (Table 2; Figure 2). Differences in trait means between the two Punkaharju subpopulations were very small: Means of Ranta-Halola and Mäkrä differed by 1 day in BST (102.7 and 103.8 days, *p* = .00019), by 1 mm in FYH (60.4 and 61.7 mm, *p* = .019), and by 0.04 in FFI (1.90 and 1.94, *p* = .016), respectively. These differences were very small relative to the within Punkaharju variation and to differences between populations.

**Table 2.** Mean and variances of the seedling traits measured in the greenhouse. For Punkaharju estimates are from model (1). For the latitudinal populations mean and total variance were calculated from the seedling data directly. Note that the proportion of seedlings not forming terminal bud during the greenhouse experiment (i.e., missing data) was largest in POL, followed by SWE, likely biasing the mean and variance of these two populations downward.

	BST 2008				FYH 2008				FFI 2010			
Pop	*n*	mean	VP	VA	*n*	Mean	VP	VA	*n*	Mean	VP	VA
PUN	11,837	103.0	83.6	40.4	11,861	60.7	250.3	141.5	5980	1.91	0.83	0.27
FINn	117	86.3	47.2	–	117	38.4	191.4	–	60	1.17	0.55	–
FINc	116	97.2	64.0	–	116	36.5	202.7	–	60	0.95	0.49	–
FINs	100	108.7	83.1	–	102	41.2	185.5	–	56	1.66	1.25	–
SWE	100	113.9	157.0	–	108	67.2	228.1	–	60	3.68	0.22	–
POL	93	114.3	210.8	–	107	72.0	260.5	–	60	3.90	0.09	–

*Note.* FINc = Central Finland; FINn = Northern Finland; FINs = Southern Finland; POL = Poland; PUN = Punkaharju; SWE = Sweden.

BST = budset timing (days); FFI = fall frost injury (score 0–4); FYH = first-year height (mm).

*n* = number of seedlings; VA = additive genetic variance; VP = total variance.

### Correlations between early seedling traits

**Figure 3.**
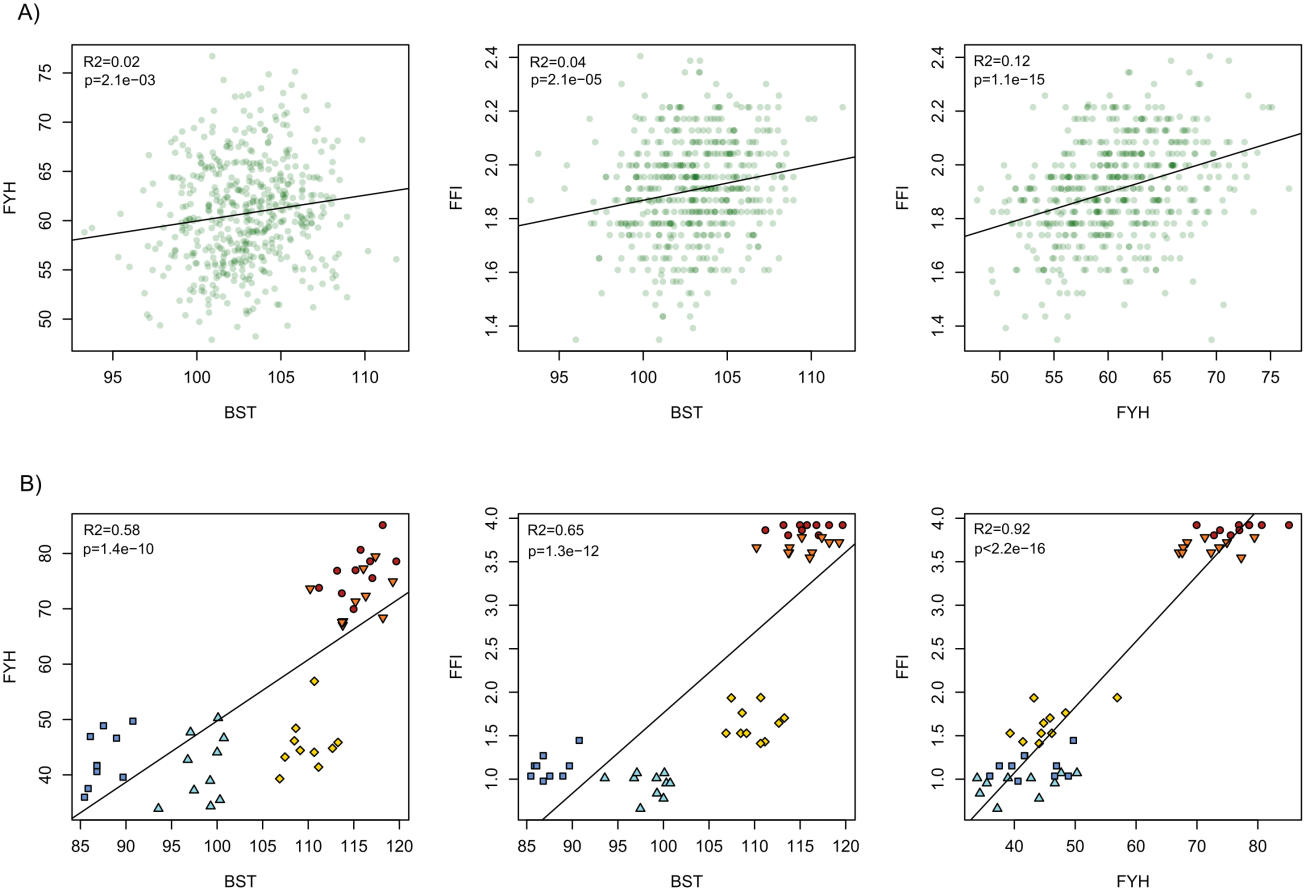
Bivariate distributions and regression lines (with coefficient of determination *R*^2^ and associated *p*-values) of seedling traits measured in the greenhouse during the first growing season. BST and FYH were measured in 2008 trial, FFI in 2010 trial. Plotted values are the family estimates (A) within Punkaharju population (*n* = 500 families), (B) in five latitudinal populations (*n* = 50 families total; circles = Poland, down-pointing triangles = Sweden, diamonds = Southern Finland, up-pointing triangles = Central Finland, squares = Northern Finland). BST = budset timing, FYH = first-year height, FFI = fall frost injury.

Between-population genetic correlations (with five latitudinal populations) varied between 0.76 and 0.99 (Table 3; Figure 3B). The within-population correlations in Punkaharju were much lower; the genetic correlations ranged from 0.14 to 0.35 (Table 3; Figure 3A). Within-population estimates of genetic correlation in the pooled data of five latitudinal populations were between 0.10 and 0.39 (Table 3). The phenotypic correlation between BST and FYH was 0.23. Low correlations were observed between seedling traits and SW, and between tree age and SW (Supplementary Figure S1). Multicollinearity was low; 1.06 (BST), 1.21 (FYH), 1.17 (FFI), and 1.08 (SW).

**Table 3.** Heritabilities and genetic correlations in seedling traits. Diagonal: Heritabilities within Punkaharju population (in bold, *n* = 500 families). Below diagonal: Genetic correlations within Punkaharju population (*n* = 500 families). Above diagonal: Genetic correlations in latitudinal populations. Between population estimates in italics: 1st line from family estimates (*n* = 50) and 2nd line from population mean of family estimates (*n* = 5). Within-population estimates (overall in five latitudinal populations) in plain text. 95% confidence intervals (for correlations) and SE (for heritabilities) in parenthesis.

	BST	FYH	FFI
BST	**0.48 (0.04)**	0.76 (0.61;0.86)*** *0.79 (−0.31;0.99)* 0.39 (0.12;0.60)**	0.81 (0.68;0.89)*** *0.83 (−0.21;0.99)* 0.10 (*−*0.18;0.37)
FYH	0.14 (0.05;0.22)**	**0.57 (0.05)**	0.96 (0.93;0.98)*** 0.99 (0.92;1.00)*** 0.39 (0.13;0.60)**
FFI	0.19 (0.10;0.27)***	0.35 (0.27;0.42)***	**0.33 (0.04)**

*Note.* BST = budset timing; FFI = fall frost injury; FYH = first-year height.

Significance from the respective Pearson’s product moment correlation: * *p* < .05, ** *p* < .01, *** *p* < .001.

### Survival and height in the field

Survival until year 2017 was followed in 4,963 Punkaharju and 295 latitudinal population seedlings. Of the Punkaharju seedlings 93% were still alive in 2010 at the time of field planting, 71% in 2011 inventory, and 64% in 2017. The respective numbers for the latitudinal populations (combined) were 73%, 42%, and 34%. The heaviest mortality took place during the first year after planting in the field. The high overall mortality within this year is likely due to random environmental factors that do not correlate strongly with the focal traits (Supplementary Tables S1 and S2).

**Figure 4.**
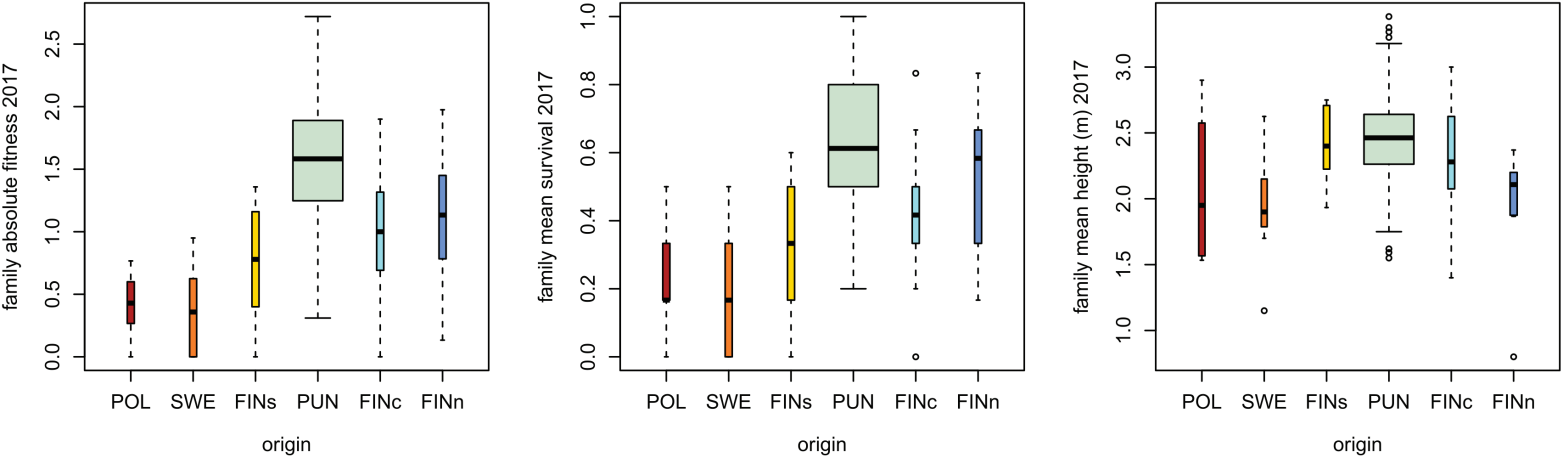
Variation in family means of fitness components measured at the field site at the age of 9 years. Family mean height is calculated only among seedlings alive in 2017. Family absolute fitness is the family mean height including also dead seedlings. POL = Poland, SWE = Sweden, FINs = Southern Finland, PUN = Punkaharju, FINc = Central Finland, FINn = Northern Finland.

In 2017, 3,165 Punkaharju seedlings were still alive. Mean height of the survivors at age of 9 years was 2.457 m (min 0.35 m; max 4.2 m). The correlation between FYH and height at 9 years was very weak (*r* = 0.063, *p* = .0005, Punkaharju only). The fitness of latitudinal populations was poorer, as predicted given local adaptation. The more southern populations had poorer survival. The northernmost population had fair survival, but the poor growth of the survivors lowered their combined fitness proxy (Figure 4).

### Selection within population

Selection gradients were calculated at the family level for BST, FYH, FFI, and SW (Table 4) to estimate selection strength and patterns. In the two-step analysis (Lande & Arnold, 1983; Phillips & Arnold, 1989), we observed statistically significant directional selection for greater FYH and stabilizing selection in FFI. Selection for positive genetic correlation between FYH and FFI was statistically significant. Selection for negative correlation between FFI and SW was statistically significant when fitness was estimated as family survival. In analysis where seedlings that died before field planting were omitted, only stabilizing selection for FFI was observed (Supplementary Table S3). In canonical analysis (Table 5), directional multitrait selection was indicated on one of the rotated axes, dominated by FYH and SW (**m**_**3**_), and stabilizing multitrait selection in another (**m**_**4**_), for which FFI contributed the most. **m**_**2**_ θ was marginally significant. The stationary point z* is within our observed range. The gradients were however very small. At the individual seedling level (with only BST and FYH, Supplementary Table S4; Supplementary Figure S2), directional selection was observed for earlier BST and greater FYH, combined with stabilizing selection for FYH.

**Table 4.** Standardized linear selection gradients (β) from the linear model and standardized quadratic and correlational selection gradients (γ) from the full quadratic model. β represents the strength of directional selection on the traits. Diagonal γ (in bold) describes the strength of stabilizing/disruptive selection on the traits. Off-diagonal γ expresses the selection on the pairwise correlation between traits. Above: gradients when family fitness was defined through both survival and height at field. Below: gradients with mean survival of the family as the fitness.

		γ			
	β	BST	FYH	FFI	SW
BST	−1.50e-03 −1.50e-02	**−2.11e-04** **−1.09e-02**	−1.48e-03 −1.45e-02	1.13e-03 1.28e-02	2.06e-03 1.23e-02
FYH	4.21e-03** 2.95e-02*		**−1.08e-03** **−2.62e-02**	3.34e-03* 3.80e-02**	−3.37e-04 −2.00e-03
FFI	−8.58e-04 −7.39e-03			−5.68e-03** −5.39e-02**	−2.80e-03. −2.88e-02*
SW	5.04e-05 4.61e-03				**8.38e-06** −**1.02e-02**

*Note.* BST = budset timing; FFI = fall frost injury; FYH = first-year height; SW = seed weight.

Significance from the respective multiple regression model: *p* < .10, * *p* < .05, ** *p* < .01, *** p < .001.

**Table 5.** Results of canonical analysis. **M** = matrix of eigenvectors from the canonical analysis, expressing the contribution of each original trait to eigenvectors. **m**_**1**_-**m**_**4**_ = eigenvectors of the response surface. θ and λ = the linear and quadratic selection gradients along the eigenvectors. z* describes the stationary point in the rotated coordinate system, min and max the observed range. Above: family fitness was defined through both survival and height at field. Below: mean survival of the family as the fitness.

	M				Selection				
	BST	FYH	FFI	SW	θ	λ	z*	min	max
**m**_**1**_	0.495 0.316	−0.474 −0.526	−0.322 −0.470	0.653 0.635	−2.45e-03 −1.19e-02	1.60e-03 9.44e-03	1.954e-06 5.616e-05	−2.378 −2.648	2.929 3.338
**m**_**2**_	0.667 0.912	0.586 0.097	0.438 0.389	0.136 −0.086	9.31e-04 −1.89e-02.	−1.76e-04 −4.09e-03	8.196e-08 −3.855e-05	−3.847 −3.383	3.193 3.400
**m**_**3**_	−0.494 −0.067	0.522 0.688	−0.156 0.142	0.677 0.709	3.63e−03** 2.91e-02**	−5.56e-04 −9.52e-03	1.009e-06 1.385e-04	−2.571 −3.139	3.538 4.193
**m**_**4**_	−0.255 −0.252	−0.398 −0.490	0.825 0.780	0.310 0.297	−2.56e-03 −2.10e-02	−4.34e-03** −4.65e-02***	-5.554e-06 -4.876e-04	−2.706 −2.737	2.155 2.042

*Note.* BST = budset timing; FFI = fall frost injury; FYH = first-year height; SW = seed weight.

Significance from the respective multiple regression model with eigenvectors: *p* < .10, * *p* < .05, ** *p* < .01, *** *p* < .001.

## Discussion

### High between-population and modest within-population correlations in seedling traits suggest mostly independent clines

Our data showed similar trend in seedling trait clines as observed in many previous studies; southern Scots pine seedlings set bud later, grew taller, and expressed more frost injuries than the northern seedlings. Concordant with the scenario of mostly independent clines, between-population correlations over five latitudinal populations were high, while the within-population correlations were lower, both in our focal Punkaharju sample, and in the pooled data of latitudinal populations.

Agreeing with our results on correlation between BST and FYH, Notivol et al. (2007) found within-population genetic correlation of 0.07 in a different set of populations of *P. sylvestris* across Europe. Mikola (1980) reported between-population correlation of 0.86 in a geographically wide sample of Scots pine populations. In Skrøppa et al. (2023), the correlation between budset forming percentage and FYH varied between −0.33 and 0.03 within Norway spruce populations. In Howe et al. (2003), see Table 3 with many tree species, within-population correlations between BST and growth range between 0.19 and 0.77 and between 0.59 and 0.86 across populations.

Hurme et al. (1997) observed very high between-population correlation between BST and frost injuries (up to 0.97) in Scots pine. Hurme et al. (2000) also found mostly independent quantitative trait loci (QTLs) for frost hardiness and BST, supporting the view of genetic independence of the traits. Within a single northern Scots pine population in a similarly designed study, Savolainen et al. (2004) reported a genetic correlation of 0.57 (SD 0.07). Our estimate was much smaller, raising the possibility that this correlation is more important in northern populations.

Generally, high between population correlations have been observed between growth and frost damage in other tree species. Within-population correlations vary from negative to positive (see Table 3 in Howe et al., 2003). In Abrahamsson et al. (2012) genetic correlation in open pollinated progeny was 0.23 (±0.20). In *Pinus contorta*, MacLachlan et al. (2021) found that many single nucleotide polymorphisms (SNPs) associated with seedling height also affected cold tolerance and phenology traits, indicating significant genetic correlation. Their study included trees from a very large geographical and environmental range.

### Field performance is consistent with local adaptation through mostly independent selection

There is ample evidence of local adaptation (Kawecki & Ebert, 2004) in Scots pine from previous studies (e.g., Eiche, 1966; Savolainen et al., 2007). Although we compared the fitness of different populations only at one site, our results were consistent with local adaptation. For both survival and height, the local population outperformed the nonlocal populations. The poor fitness of the southern populations was dominated by low survival, likely due to lower cold tolerance and later cessation of growth (Supplementary Tables S1 and S2). Northernmost population had high survival, but poorer height.

The Lande–Arnold analysis showed statistically significant directional selection for greater FYH and stabilizing selection for FFI, although with very modest gradients, and some indications for correlated selection. A closer look at the trait combinations with canonical analysis revealed directional selection on the rotated axis **m**_**3**_ reflecting the higher fitness of tall seedlings, to which large seed weight also contributed. Stabilizing selection was observed on **m**_**4**_, where FFI contributes the most.

Weaker selection gradients from reanalysis without seedlings that died already before field planting highlighted the importance of early selection (Supplementary Table S3). Luoranen et al. (2018) showed that most of the damage observed after 1 year after planting was due to frost. General vigor of seedlings and interactions with other factors such as drought may also contribute. Early selection is important also in *P. contorta*, related to drought (Warwell & Shaw, 2019). However, there is also much early random mortality (Lönnroth, 1925; Sarvas, 1962).

Among plant functional traits, there is often selection for earlier phenology (toward earlier and shorter flowering and rapid germination) and for increased size (Caruso et al., 2020). Liu and El-Kassaby (2019) observed selection for later BST in *Populus trichocarpa*. At the family level we did not observe clear signs of selection for BST. In the canonical analysis **m**_**2**_—to which BST had the highest contribution—had a marginally significant θ. At the individual seedling level Supplementary Table S4; Supplementary Figure S2) earlier budset was favored but cold damage data were not available for the same seedlings. Thus, the measured selection on BST might have been due to the low correlation with FFI. For FYH, genetic- and phenotypic-level analyses agree on the directional selection, although the selection seemed much stronger at the phenotypic level, as the range of variation was much larger than for family estimates.

Although our selection analysis showed statistically significant multitrait selection, the coefficients were small. This is in line with the rather low within-population genetic correlation we observe; strong selection favoring a specific combination of traits would be expected to strengthen the genetic correlation (Roff & Fairbairn, 2012; Svensson et al., 2021).

### Modest selection allows maintenance of large within-population variation within a latitudinal cline

In genetic models of clinal variation, stabilizing selection operates within populations to keep them close to their local optima (Barton, 1999; Slatkin, 1978). The lack of stabilizing selection on BST and FYH here contrasts with this expectation. However, 9-year-old trees have experienced only a part of the selection as selection pressures can vary within one generation (e.g., Grant & Grant, 1995; Kelly, 1992; Siepielski et al., 2009). Selection due to climate acts early (Haig et al., 1941; Leck et al., 2008), which could favor cold adapted genotypes, whereas competition, possibly favoring taller trees, acts later when the trees are large enough to interfere with each other, for example, by shading or nutrient competition. In a field study with higher density, canopy closure took place at age of 11 and lead to increased mortality in Scots pine (Koelewijn et al., 1999, see also Egbäck et al., 2012). Observing larger selection gradients when using only family mean survival as fitness measure supports this view.

Earlier studies agree with our data showing that despite the pronounced cline, variation in seedling traits exists within Scots pine populations (Hurme et al., 1997; Kujala et al., 2017; Mikola, 1982; Notivol et al., 2007; Savolainen et al., 2004). Pollen-driven gene flow and recombination are expected to generate the extensive variation. Within-population selection cannot be strong relative to gene flow, congruent with fairly high heritability estimates in this and previous studies (Wheelwright et al., 2014). Nevertheless, earlier reciprocal transplant experiments show that even rather short north/south transfers result in fitness reduction (Berlin et al., 2016; Eiche, 1966; Savolainen et al., 2007). With different optima along the cline, strong within-population stabilizing selection is not a necessary condition for high between-population differentiation (*Q*_ST_) and cline formation even under strong gene flow (see simulation work of Le Corre & Kremer, 2003, Figure 6).

### Parallel clines in changing climate

In northern latitudes, the climate is warming rapidly. The growing season in Scandinavia has increased by ~1 week between 1951 and 2000 and is expected to increase by 20–30 days within few decades in most areas in Finland and in northern and middle Sweden (Bärring et al., 2017; Linderholm et al., 2008). However, photoperiodic conditions will remain the same. As seedling traits in northern populations of Scots pine are adapted to local combinations of photoperiod and temperature (Koski & Sievänen, 1985; Oleksyn et al., 1992, 1998; Olsen, 2010; Vaartaja, 1959), a genetic change will be needed for growth to continue longer while ensuring correct timing of dormancy (Hänninen et al., 2001; Saikkonen et al., 2012; Savolainen et al., 2004, 2011; Singh et al., 2017).

Genetic correlations can either reinforce or impede evolutionary responses, depending on the direction of correlation in relation to selection (Lande, 1979). In the legume *Chamaecrista fasciculata*, correlations likely will constrain adaptation to climate change (Etterson & Shaw, 2001). MacLachlan et al. (2021) suggested that shared variation between adaptive traits in *P. contorta* will result in correlated changes. In Scots pine with largely independent clines, adaptation can be flexible under changing conditions (e.g., de La Mata et al., 2017). Furthermore, high heritabilities in adaptive traits, combined with southern gene flow, will contribute to the adaptive capacity. Similar amounts of genetic variation likely exist in other parts of the clines, too, with a potential exception of range edges (Eckert et al., 2008; Kujala & Knürr et al., 2017).

This analysis concentrated on the capacity for genetic adaptation. To predict future responses, it will be necessary to also consider phenotypic plasticity (e.g., Benito Garzón et al., 2011; Hämälä et al., 2022), effects of demography, such as high survival of old trees limiting establishment possibilities (Kuparinen et al., 2010; Savolainen, 2004), and competition and other interactions with other species. Here our main findings come from a single population, and more empirical data should be obtained from other populations to verify the genetic independence of the seedling traits in other parts of the cline. However, this highly powered analysis of one large population already shows that correlated selection alone has not generated the parallel clines.

Current clines show that historically, genetic adaptation has been a very important component of responses to environmental changes (Davis & Shaw, 2001). Increasing information on the genetics of local adaptation in natural populations of forest trees will enable mitigation of climate change effects in artificially regenerated forests, too, via breeding for new conditions and determining use of regeneration material for changing climate (Aitken et al., 2008; Berlin et al., 2016; Cortés et al., 2020; MacLachlan et al., 2017).

## Supplementary Material

qrad054_suppl_Supplementary_Figures_S1-S2_Tables_S1-S4

## Data Availability

Data and R commands have been deposited to DRYAD (https://doi.org/10.5061/dryad.vmcvdnd09).
