# Supplementary material for: Within- and between-population comparisons suggest independently acting selection maintaining parallel clines in Scots pine (Pinus sylvestris)
Source: Evol Lett. 2023 Nov 27;8(2):231–42. doi: 10.1093/evlett/qrad054 (PMC10959485; doi:10.1093/evlett/qrad054)
Supplement: qrad054_suppl_Supplementary_Figures_S1-S2_Tables_S1-S4 [file qrad054_suppl_supplementary_figures_s1-s2_tables_s1-s4.pdf]

Supplementary Material: Maternal effects

We also examined the effect of seed weight and the mother tree's age on the seedling traits. This was done at the family level as Pearson correlation, using seed lot weight for each mother tree. A dependency was observed between FYH and seed weight; mother trees with heavier seeds produce somewhat taller seedlings (r=0.23, p=1.279e-07). Small correlation between FFI and seed weight (r=0.12, p=0.007019) is likely indirect, through their associations with FYH. Even smaller effect of seed weight was observed on BST (r=-0.093, p=0.0371).

Younger mother trees have slightly taller seedling (r=-0.17, p= 0.000185). This can be due to the fact that seed weight is slightly lower among older trees (r=-0.24, p=1.093e-07). We further checked whether this correlation arises from spatial covariation between the age of the mother tree and a hypothetical environmental factor affecting seed weight. Spatial autocorrelation in tree age, seed weight, and in the residuals of a linear model of weight vs. age was also explored in the Ranta-Halola forest from the plots of the omnidirectional empirical variograms, computed using the gstat package (Pebesma 2004, Version 2.0-6) in the R environment (R Core Team 2020, Version 4.0.4). Adjusting the recommendation of Journel and Huijbregts (1978, p. 194) for the distinctly non-square region, semivariances were computed for 20 distance bins of equal length extending up to half the maximum difference of the y-coordinates.

Although a significant spatial autocorrelation was found for tree age, no such structure was evident in seed weights or in the model residuals, confirming that the correlation between age and seed weight was not due to spatial environmental effects.

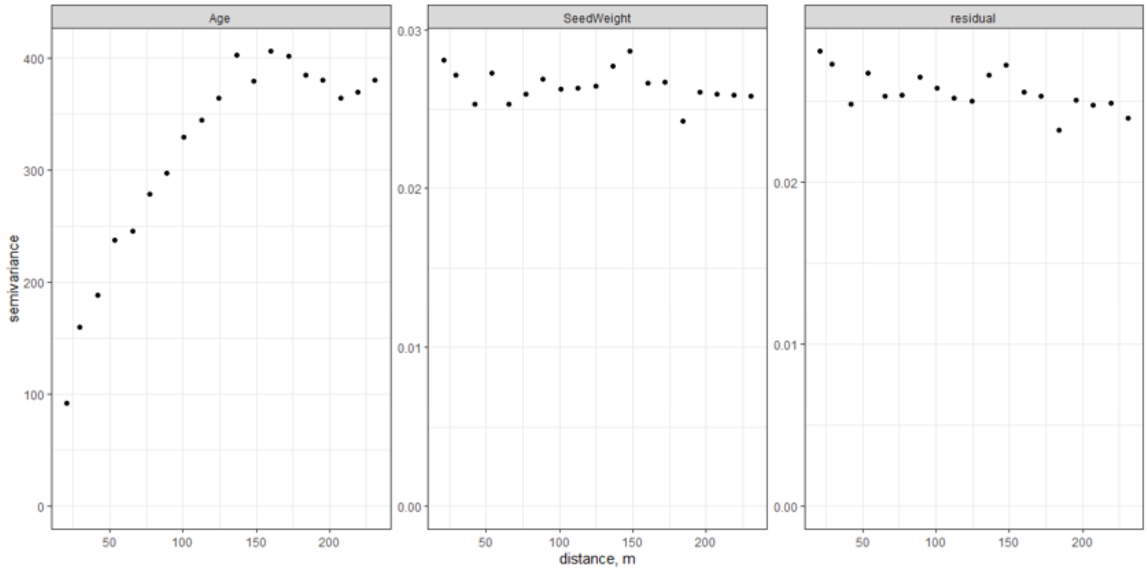

Figure S1. Variograms showing spatial autocorrelation in tree age, seed weight, and in the residuals of a linear model of weight vs. age in the Ranta-Halola subpopulation.

The positive effect of seed weight on seedling height in early years has been reported many times (e.g., Mikola 1985, Wennström et al. 2002, Reich et al. 1994). In Maritime pine heritable genetic variation was observed for seed weight (Zas & Sampedro 2015). Ramirez-Valiente et al. 2020 found evidence for selection for larger seed mass in an analysis where multiple populations from different latitudinal origin were combined to estimate selection. This dependency should be kept in mind when interpreting selection effects on FYH.

Considering the speed of current environmental change, the long generation time of Scots pine obviously slows down adaptation (e.g., Savolainen et al., 2004). The negative correlation between mother tree’s age and first year seedling height, can result from younger trees having slightly heavier seed. Through the observed selective advantage of tall seedlings, alleles contributed by young trees could have some competitive advantage. This association might have some influence on shortening the generation time and accelerating adaptation.

References:

Journel, A. G., and Huijbregts, C. J. (1978). Mining Geostatistics. New York: Academic Press

Mikola, J. 1985. Relationships between height growth differences of Scots pine full-sib families and variation in seed size, annual growth rhythm, and some foliage characteristics. In Tigerstedt, P. M. A., Puttonen, P. & Koski, V. (eds). Proceedings of an International Conference on Managing Forest Tree as Plants, Crop Physiology of Forest Trees, 23–28 July 1984, Helsinki, Finland, pp. 233–243. ISBN 951-45-3705-X

Pebesma, E.J. (2004). Multivariable geostatistics in S: the gstat package. Computers & Geosciences, 30: 683-691.

Ramírez-Valiente, J. A., Solé-Medina, A., Pyhäjärvi, T., Savolainen, O., Cervantes, S., Kesälahti, R., Kujala, S. T., Kumpula, T., Heer, K., Opgenoorth, L., & others. (2021). Selection patterns on early-life phenotypic traits in Pinus sylvestris are associated with precipitation and temperature along a climatic gradient in Europe. New Phytologist, 229(5), 3009–3025. https://doi.org/10.1111/nph.17029

R Core Team (2020). R: A language and environment for statistical computing. R Foundation for Statistical Computing, Vienna, Austria. URL https://www.R-project.org/.

Reich, P. B., Oleksyn, J., & Tjoelker, M. G. (1994). Seed mass effects on germination and growth of diverse European Scots pine populations. Canadian Journal of Forest Research, 24(2), 306-320.

Savolainen, O., Bokma, F., Garća-Gil, R., Komulainen, P., & Repo, T. (2004). Genetic variation in cessation of growth and frost hardiness and consequences for adaptation of Pinus sylvestris to climatic changes. Forest Ecology and Management, 197(1–3), 79–89. https://doi.org/10.1016/j.foreco.2004.05.006

Wennström, U., Bergsten U. & Nilsson J.-E. (2002) Effects of Seed Weight and Seed Type on Early Seedling Growth of Pinus sylvestris under Harsh and Optimal Conditions. Scandinavian Journal of Forest Research, 17(2), 118-130, DOI:10.1080/028275802753626764

Zas, R., & Sampedro, L. (2015). Heritability of seed weight in Maritime pine, a relevant trait in the transmission of environmental maternal effects. Heredity, 114(1), 116-124.

Supplementary Material: Early effects of selection

Here we examined in more detail the mortality/selection effects on the very first years of the seedlings lives. We first compared the distributions of BST (budset timing) and FYH (first year height) between groups of seedlings scored dead and alive at different timepoints (at 2010 inventory, at 2011 inventory, and between these two inventories). Each population was analyzed separately. Differences in group means were tested with Welch Two Sample (two-sided) t-test.

We found that by 2010, in the southernmost population samples, there was a trend of surviving plants to be shorter and have earlier budset than those that died, consistent with selection by frosts. In the focal population and other Finnish populations, the survivors were taller, suggesting general vigor. During the year after planting (2010-2011), the trend was mostly similar. The high overall mortality within this year is likely due to random environmental factors that do not correlate strongly with the focal traits (see Lönnroth 1925).

Table S1. Welch Two Sample (two-sided) t-test for differences between the means of budset timing (BST) of dead and live seedlings at different timepoints. Population means were calculated from individual seedling data. The p-values are not corrected for multiple testing.

	by 2010			2010-2011			by 2011		
	x	y	p-value	x	y	p-value	x	y	p-value
PUN	102.6	102.1	0.397	102.0	102.1	0.638	102.1	102.1	0.961
FINn	83.4	86.0	0.228	85.5	86.3	0.724	84.5	86.3	0.346
FINc	95.1	95.9	0.680	94.9	96.4	0.624	95.0	96.4	0.537
FINs	109.4	107.9	0.620	109.8	106.8	0.363	109.5	106.8	0.346
SWE	118.0	115.8	0.449	117.8	113.3	0.165	117.8	113.3	0.148
POL	121.1	113.9	0.049	112.3	116.1	0.359	114.8	116.1	0.702

x = mean of dead seedlings
y = mean of live seedlings

Table S2. Welch Two Sample (two-sided) t-test for differences between the means of first year height (FYH) of dead and live seedlings at different timepoints. Population means were calculated from individual seedling data. The p-values are not corrected for multiple testing.

	by 2010			2010-2011			by 2011		
	x	y	p-value	x	y	p-value	x	y	p-value
PUN	51.4	58.5	6.8e-11	56.4	59.1	4.6e-07	55.1	59.1	8.2e-15
FINn	28.4	39.5	7.9e-03	37.6	40.3	0.498	33.4	40.3	0.044
FINc	30.8	38.3	0.033	36.5	39.1	0.606	33.0	39.1	0.082
FINs	27.9	41.8	9.5e-05	35.1	45.3	0.075	30.3	45.3	3.3e-04
SWE	68.2	64.1	0.362	66.4	60.8	0.216	67.0	60.8	0.163
POL	75.9	72.6	0.516	72.2	72.1	0.992	73.4	72.1	0.776

x = mean of dead seedlings
y = mean of live seedlings

We also took a closer look at the relative importance of selection during the very first years versus later years. For this, we repeated the two-step Lande-Arnold analysis (Lande & Arnold, 1983; Phillips & Arnold, 1989, see main text) by omitting seedlings that died before planting in the field. We found that the signals of selection were weaker with this subdata, indicating that selection on these traits acts rather early. Young Scots pine seedlings are susceptible to frost damage at early ages (Luoranen et al. 2018) and importance of early selection has been found also e.g. in *Pinus contorta* (Warwell & Shaw, 2019).

Table S3. Standardized linear selection gradients (β) from the linear model and standardized quadratic and correlational selection gradients (γ) from the full quadratic model, excluding seedlings that died already before planting to field (dead in 2010 inventory). β represent the strength of directional selection on the traits. Diagonal γ (in bold) describe the strength of stabilizing/disruptive selection on the traits. Off-diagonal γ express the selection on the pairwise correlation between traits. Above: gradients when family fitness was defined through both survival and height at field. Below: gradients with mean survival of the family as the fitness.

		γ			
	β	BST	FYH	FFI	SW
BST	-5.67e-05<br>1.59e-04	**1.79e-04**<br>**-7.56e-03**	-9.76e-04<br>-8.92e-03	4.01e-04<br>6.58e-03	1.67e-03<br>5.06e-03
FYH	2.26e-03<br>1.71e-02		**1.45e-03**<br>**-2.49e-03**	8.23e-04<br>2.47e-02	-1.35e-03<br>-1.39e-02
FFI	-4.13e-04<br>-4.45e-03			**-3.15e-03**<br>**-4.12e-02***	-8.98e-04<br>-1.07e-02
SW	8.67e-04<br>1.39e-02				**6.55e-04**<br>**-1.61e-03**

BST = budset timing; FYH = first year height; FFI = fall frost injury; SW = seed weight.
Significance from the respective multiple regression model: . P<0.10, * P < 0.05, ** P < 0.01, *** P < 0.001.

References:

Lande, R., & Arnold, S. J. (1983). The measurement of selection on correlated characters. Evolution,1210–1226. https://doi.org/10.2307/2408842

Luoranen, J., Saksa, T., & Lappi, J. (2018). Seedling, planting site and weather factors affecting the success of autumn plantings in Norway spruce and Scots pine seedlings. For Ecol Manag 419–420: 79–90.

Lönnroth, E. (1925) Untersuchungen über die innere Struktur und Entwicklung gleichaltriger naturnormaler Kiefernbestände: basiert auf Material aus der Südhälfte. Acta Forestalia Fennica, 269, 30.

Phillips, P. C., & Arnold, S. J. (1989). Visualizing multivariate selection. Evolution, 43(6), 1209–1222.

Warwell, M. v., & Shaw, R. G. (2018). Phenotypic selection on growth rhythm in whitebark pine under climatic conditions warmer than seed origins. Journal of Evolutionary Biology, 31(9), 1284–1299. https://doi.org/10.1111/jeb.13301

Supplementary Material: Selection analysis with aster at the individual seedling level

We calculated selection gradients at the phenotypic (individual seedling) level with the Aster method (Geyer et al., 2007) within Punkaharju. Only BST (budset timing) and FYH (first year height) where included. FFI (fall frost injury) was estimated from different seedlings, albeit in the same families, and could thus not be included. SW (seed weight) was measured only at the family level. The R package aster (Geyer, 2021, version 1.1-2) was used. Aster allows using multiple hierarchical fitness components in the analysis. The interdependencies and different distribution families of the fitness components are specifically defined in the model. Our basic aster model was simple, for each seedling:

(Root) → Survival 2010 → Survival 2017 → Height 2017

where survival 2010 refers to the inventory done at the stage of planting the seedlings in the field, and 2017 survival and height refer to the status of the seedling at the age of nine years. Root was set to 1, survival 2010 and 2017 were modeled as Bernoulli (0 for dead, 1 for alive), and height 2017 as Gaussian (with standard deviation estimated from the data, excluding dead seedlings). Survival in 2011 was not taken as a node, as there were more missing data in that inventory. Hierarchical absolute fitness as dependent variable was modeled with linear and quadratic effects for both BST and FYH and with their interaction. The predictor variables were first standardized to have a mean of zero and unit variance. Greenhouse block and field block were included as fixed effects. Seedlings with missing values in any of the traits or fitness components were omitted. A total of 4807 seedlings were included. The significance of the block terms was tested with the anova function built in the aster package by dropping the respective term from the model and comparing this reduced model against the full model. Regression coefficients from the full model were taken as the estimates of beta (coefficients

for linear terms for directional selection) and gamma (coefficients for quadratic terms for stabilizing and correlational selection). Estimates for stabilizing selection were doubled (Stinchcombe et al. 2008). To evaluate the relative importance of survival versus tree height as a fitness estimator, we also tested a model excluding the tree height:

(Root) → Survival 2010 → Survival 2017

Further, we ran the full model with a subset of data (n=4463, omitting seedlings that died before field planting) to examine the significance of very early selection:

(Root) → Survival 2017 → Height 2017

Directional selection was found for earlier BST and greater height (table S4, fig. S1). However, the very tallest seedlings did not have the highest fitness, resulting in stabilizing selection in FYH. When only survival was considered, the results were similar, but the coefficients were generally larger. When using only seedlings still alive at 2010 inventory/field planting (i.e., omitting selection on the first, seedling nursery years), directional selection on both traits was slightly weaker and no stabilizing selection was detected. The greenhouse block effect was statistically significant when early survival was included in the analysis, but not in later years. The field block effect was statistically significant when tree height was included in the fitness estimate, but not when only survival was considered. Both block terms were however kept in all models for the sake of model comparison. The gamma term for interaction (i.e., correlated selection) was not statistically significant in any analyses.

At the family level (see main text) we did not observe clear signs of selection for timing of budset, nor stabilizing selection for FYH. The aster results here could thus be influenced by unobserved selection in FFI and SW. This result, however, is concordant with a hypothesis of

southern late (and cold prone) alleles being selected against in the seedling population (see e.g., García-Ramos & Kirkpatrick, 1997). Southerly winds prevailing at the time of pollination could introduce nonlocal alleles, as female flowers can be receptive before local pollen shedding (Sarvas, 1962; Varis et al., 2009; see also Kling & Ackerly, 2021).

Table S4. Selection gradients from the Aster analyses. a) full model, all Punkaharju seedlings (n=4807); b) survival as the terminal node, all Punkaharju seedlings (n=4807); c) full model, excluding seedlings that died before 2010 (n=4463).

	a)	b)	c)
βBST	-4.46e-02***	-1.03e-01**	-3.15e-02*
βFYH	1.05e-01***	2.48e-01***	7.16e-02***
γBST BST	-9.69e-04	6.10e-03	7.67e-03
γFYH FYH	-7.44e-02***	-1.93e-01***	-2.13e-02
γBST FYH	-1.59e-02	-3.01e-02	-1.28e-02

Selection gradients: βBST = directional selection in timing of bud set; βFYH = directional selection in first year height; γBST BST = stabilizing selection in timing of bud set; γFYH FYH = stabilizing selection on first year height; γBST FYH = selection on correlation between timing of bud set and first year height.

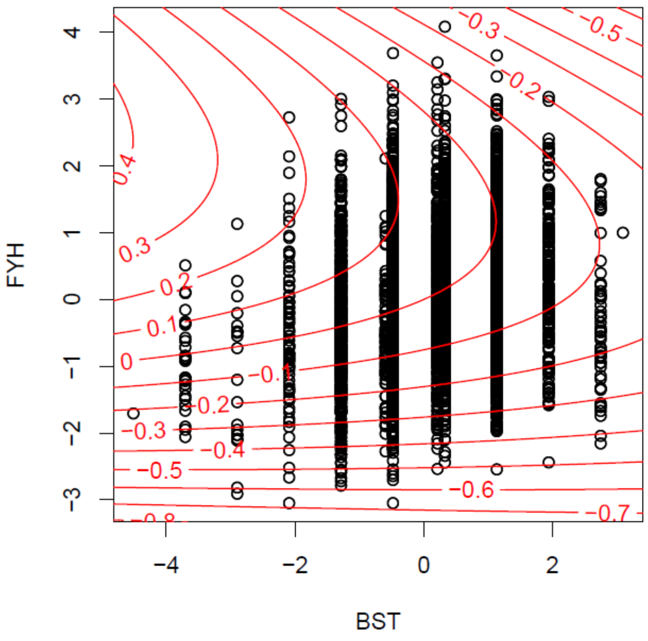

Figure S2. A graphical representation of selection on BST and FYH. The dots are the actual measures of the traits in the greenhouse. The contour lines represent the approximation of the individual selection surface from the aster analysis. Highest fitness is found among seedlings that are taller than average (but not the tallest) and have early budset timing.

References:

García-Ramos, G., & Kirkpatrick, M. (1997). Genetic models of adaptation and gene flow in 523 peripheral populations. Evolution, 51(1), 21–28.

Geyer, C. J. (2021). aster: Aster Models. https://CRAN.R-project.org/package=aster

Geyer, C. J., Wagenius, S., & Shaw, R. G. (2007). Aster models for life history analysis. Biometrika, 94(2), 415–426. https://doi.org/10.1093/biomet/asm030

Kling, M. M., & Ackerly, D. D. (2021). Global wind patterns shape genetic differentiation, asymmetric gene flow, and genetic diversity in trees. Proceedings of the National Academy of Sciences, 118(17), e2017317118.

Sarvas, R. (1962). Investigations on the flowering and seed crop of Pinus Silvestris. Communicationes 646 Instituti Forestalis Fenniae, 53(4), 1–198.

Stinchcombe, J. R., Agrawal, A. F., Hohenlohe, P. A., Arnold, S. J., & Blows, M. W. (2008). Estimating nonlinear selection gradients using quadratic regression coefficients: double or nothing? Evolution, 62(9), 2435-2440. https://doi.org/10.1111/j.1558-5646.2008.00449.x

Varis, S., Pakkanen, A., Galofré, A., & Pulkkinen, P. (2009). The extent of south-north pollen transfer 680 in Finnish Scots pine. Silva Fennica, 43(5), 717–726. https://doi.org/10.14214/sf.168
